# Physiological and proteomic analyses revealed the response mechanisms of two different drought-resistant maize varieties

**DOI:** 10.1186/s12870-021-03295-w

**Published:** 2021-11-04

**Authors:** Hongjie Li, Mei Yang, Chengfeng Zhao, Yifan Wang, Renhe Zhang

**Affiliations:** College of Agronomy, Northwest A&F University, Yangling, 712100 Shaanxi China

**Keywords:** Drought tolerance, iTRAQ, Maize, Photosynthesis

## Abstract

**Background:**

Drought stress severely limits maize seedling growth and crop yield. Previous studies have elucidated the mechanisms by which maize acquires drought resistance and contends with water deficiency. However, the link between the physiological and molecular variations among maize cultivars are unknown. Here, physiological and proteomic analyses were conducted to compare the stress responses of two maize cultivars with contrasting drought stress tolerance.

**Results:**

The physiological analysis showed that the drought-tolerant SD609 maize variety maintains relatively high photochemical efficiency by enhancing its protective cyclic electron flow (CEF) mechanism and antioxidative enzymes activities. Proteomics analysis revealed that 198 and 102 proteins were differentially expressed in SD609 and the drought-sensitive SD902 cultivar, respectively. GO and KEGG enrichments indicated that SD609 upregulated proteins associated with photosynthesis, antioxidants/detoxifying enzymes, molecular chaperones and metabolic enzymes. Upregulation of the proteins related to PSII repair and photoprotection improved photochemical capacity in SD609 subjected to moderate drought stress. In SD902, however, only the molecular chaperones and sucrose synthesis pathways were induced and they failed to protect the impaired photosystem. Further analysis demonstrated that proteins related to the electron transport chain (ETC) and redox homeostasis as well as heat shock proteins (HSPs) may be important in protecting plants from drought stress.

**Conclusions:**

Our experiments explored the mechanism of drought tolerance and clarified the interconnections between the physiological and proteomic factors contributing to it. In summary, our findings aid in further understanding of the drought tolerance mechanisms in maize.

**Supplementary Information:**

The online version contains supplementary material available at 10.1186/s12870-021-03295-w.

## Background

Maize (*Zea mays* L.) is an important cereal crop used as food, feed and fuel. It is subjected to different types of abiotic stress throughout its life cycle [1]. Water resources shortage has become a major challenge in agricultural production and social development in the arid regions of northwestern China [2]. As the main grain crop in this region, significant portions of the maize suffer from drought-induced yield losses. Thus, greater yield stability via improved drought tolerance is a priority objective of maize breeders [3]. Plant geneticists have used a wide range of technologies to develop crop varieties that perform well under drought stress conditions. Therefore, understanding the drought tolerance mechanisms in resistant maize varieties is vital to genetic manipulation and/or cross breeding in maize.

The responses of plants to drought stress are highly complex, especially chloroplast metabolism. Photosynthesis, the most fundamental process, is severely affected by water stress [4]. Under drought stress, photosynthetic activity is disturbed due to chlorophyll degradation, stomatal closure, inhibition of enzymes (such as Rubisco) and diminished photochemical efficiency of Photosystem II (PSII) [5]. Down regulation of PSII activity will result in an imbalance between the light absorption and utilization [6]. Excess light energy generates reactive oxygen species (ROS) such as O_2_^−^, ^1^O_2_, H_2_O_2_, OH, which are potentially detrimental and can inhibit the repair of PSII [7]. Tolerant genotypes have highly active enzymatic superoxide dismutase (SOD), peroxidase (POD), catalase (CAT), glutathione reductase (GR) and non-enzymatic (carotenoids and anthocyanins) antioxidant systems [6]. Over the past years, major research efforts have focused on the various of physiological, biochemical, molecular and transcriptome analysis [8–12]. Nevertheless, transcriptome-level drought tolerance studies cannot fully elucidate the regulatory mechanism of drought tolerance. Furthermore, transcript abundance usually are not in accordance with the protein abundance and physiological performance [13]. Consequently, comprehensive information is lacking regarding the interactions among the processes regulating drought stress in maize.

Proteomics is a powerful tool providing overviews of the cellular and molecular changes that occur in plants under drought stress. Two-dimensional electrophoresis (2-DE) and two-dimensional differential gel electrophoresis (2D-DIGE) have been widely applied in differential proteome analyses of wheat [14] and rice [15] under stress conditions. However, there are several inherent limitations in 2D-gel-based approaches. These include low protein identification rates, low reproducibility and difficulty in separating low-molecular-weight proteins [16, 17]. Isobaric tags for relative and absolute quantification (iTRAQ) can identify many different proteins and provide more reliable quantitative information than 2-DE analyses [18]. Proteomic studies on drought stress have focused mainly on inbred maize [19–21]. Nevertheless, the combination of iTRAQ-based quantitative proteomics and detailed physiological analyses has seldom been implemented to study the response mechanisms of plants, and specifically maize, under drought stress.

In this work, we took two hybrids maize cultivars, drought-tolerant (Shaandan 609) and drought-sensitive (Shaandan 902), as materials and systematically compared their physiological responses under moderate drought stress. We measured their leaf chlorophyll content, photosynthetic energy efficiency, and antioxidant enzyme activities. We also compared their protein expression levels by iTRAQ tandem mass spectrometry (MS/MS) technology and experimentally verified these results by qRT-PCR. The aims of the present study were to: (i) perform physiological and proteomic analyses to compare the response mechanisms of drought-tolerant and drought-sensitive maize cultivars, and (ii) correlate the physiological and proteomic data and link the molecular and physiological responses of maize under drought stress. The results of this study will help clarify drought tolerance mechanisms in maize and facilitate the development of novel maize cultivars with superior water use efficiency.

## Results

### Changes in the phenotypic and photosynthetic parameters of two maize varieties under moderate drought stress

**Fig. 1:**
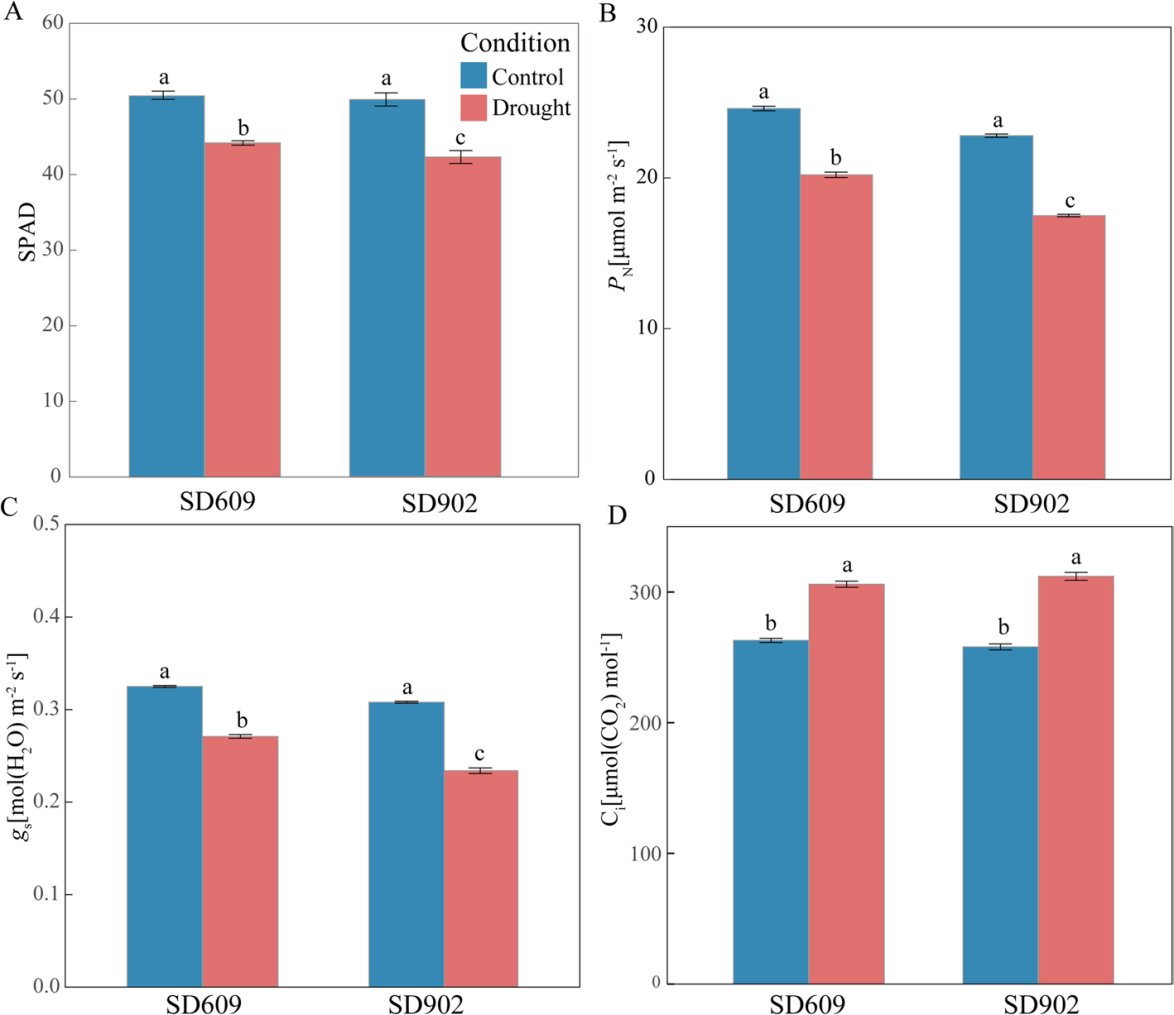
Changes in chlorophyll content and gas change of two maize varieties under moderate drought. **A** Total chlorophyll content (SPAD), **B** Net photosynthetic rate (*P*_N_), **C** Stomatal conductance (*g*_s_), **D** Substomatal CO_2_ concentration (*C*_i_). All data represent means ± standard errors of three replicates. Values with different letters indicate significant difference at *P* < 0.05 level between treatments based on one-way ANOVA

Plants under moderate drought stress presented with significantly lower net photosynthetic rate (*P*_N_) and stomatal conductance (*g*_s_) and significantly higher intercellular CO_2_ concentration (*C*_i_) than the control plants. As shown in (Fig. 1b, c, d), the changes in all parameters in SD902 were higher than SD609. Compared with the control, the intercellular CO_2_ concentrations in SD609 and SD902 had increased by 16.3 and 20.9%, respectively.

**Fig. 2:**
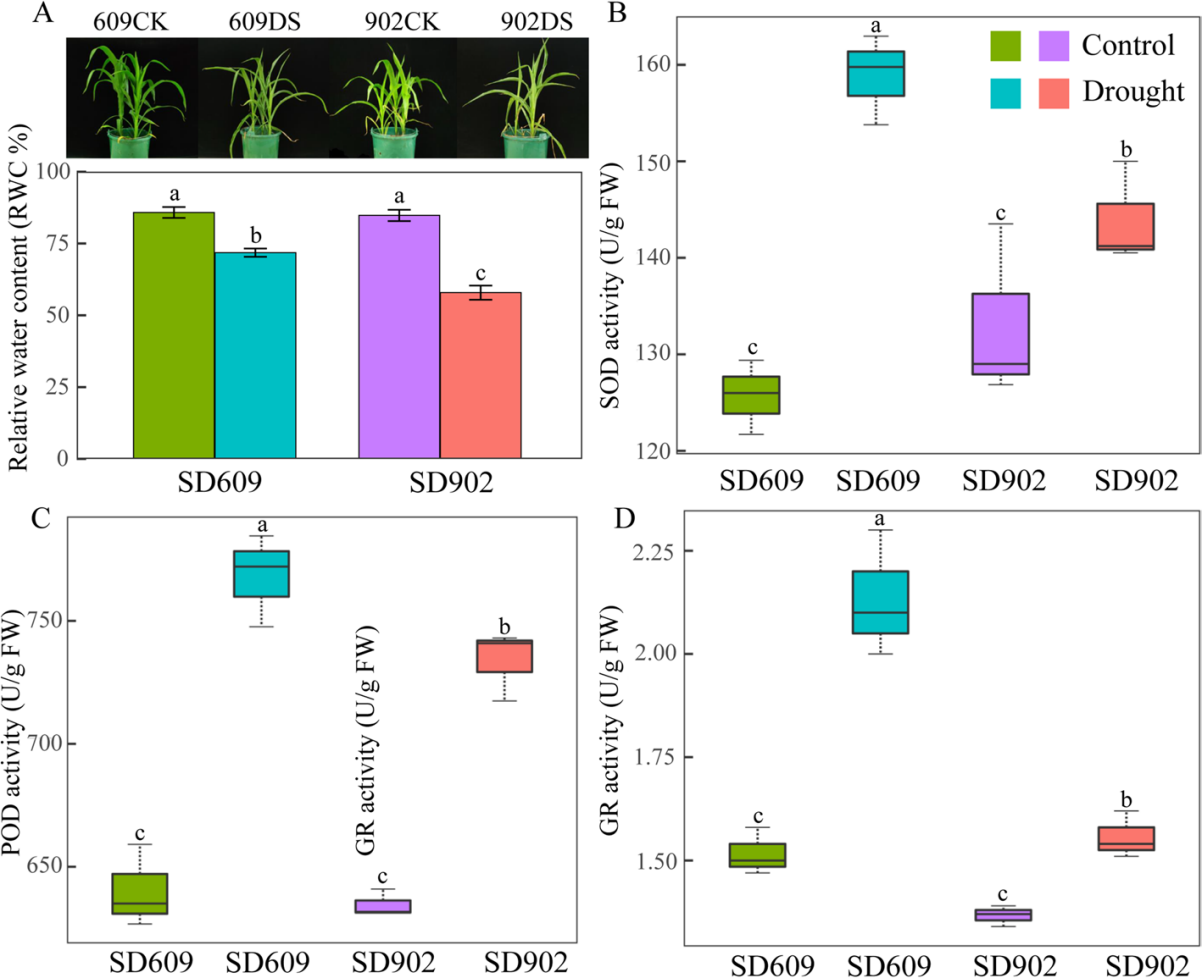
Morphological and antioxidant activities of SD609 and SD902 in response to drought stress. **A** Growth and the leaf relative water content (RWC) of two maize varieties under well-watered (CK) and moderate-drought (DS) stress conditions. **B** Superoxide dismutase (SOD) enzyme activity. **C** Peroxidase (POD) enzyme activity. **D** Glutathione reductase (GR) enzyme activity. All data represent means ± standard errors of three replicates. Values with different letters indicate significant difference at *P* < 0.05 level between treatments based on one-way ANOVA

The phenotypic responses to drought stress widely differed between the two varieties. Under moderate drought stress, the leaf tips of SD902 curled more severely than those of SD609, and the seedlings of SD609 seedlings appeared greener than the SD902 seedlings (Fig. 2a). Moreover, the chlorophyll content (SPAD) and RWC were higher in SD609 than SD902 under both control and water deficit conditions (Fig. 1a). These findings were consistent with those of the visual observations. Relative to the control, RWC levels decreased in SD609 and SD902, under drought stress by 16.3 and 31.7%, respectively.

### Changes in the photosynthetic parameters and protective enzymes of two maize varieties under moderate drought stress

**Fig. 3:**
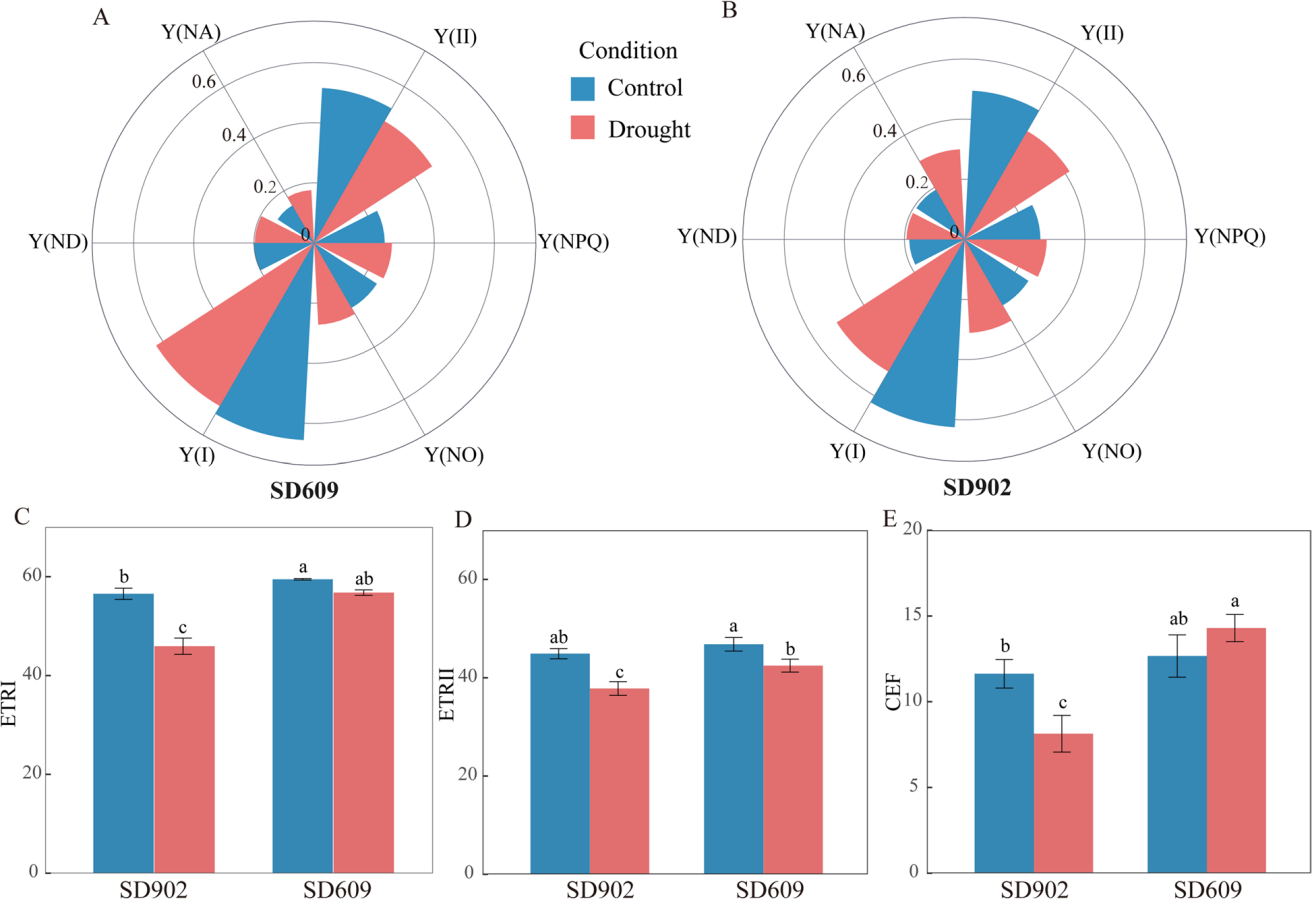
Changes of photochemical efficiency of two maize varieties under drought stress. **A** Drought induced changes in PSII and PSI parameters of SD609. Y(II), the effective quantum yield of PSII photochemistry; Y(NPQ), the quantum yield of PSII regulated nonphotochemical energy dissipation; Y(NO), the quantum yield of nonregulated nonphotochemical energy dissipation; Y(I), the effective quantum yield of PSI photochemistry; Y(NA), the nonphotochemical quantum yield of the PSI acceptor side. Y(ND), the nonphotochemical quantum yield of the PSI donor side. **B** Drought induced changes in PSII and PSI parameters of SD902. **C** ETRI, electron transport rate through PSI. **D** ETRII, electron transport rate through PSII. **E** CEF activity of SD609 and SD902 under moderate drought stress. Each parameter represents means ± standard errors of three replicates. The different letters above the columns indicate significant difference at *P* < 0.05 level between treatments

Drought stress significantly altered photosynthetic efficiency based on the energy conversion in PSII and PSI. As shown in Fig. 3a, b, the value of Y(II) and Y(I) under drought stress were lower than under control conditions in two varieties, especially in SD902. The decline in Y(II) was accompanied by the increase of Y(NPQ) and Y(NO) in both varieties, of which Y(NO) increased significantly in SD902. Changes in Y(NA) and Y(ND) accompanied the increase in Y(I) and these changes were higher in SD902 than SD609 (Fig. 3a, b). We determined Y(CEF) for both varieties under moderate drought stress by estimating ETRI and ETRII (Fig. 3c, d). ETRI and ETRII were significantly lower under moderate drought stress than control conditions. There was a clear difference between varieties in terms of Y(CEF) (Fig. 3e). Compared with the control, Y(CEF) was higher in SD609 and lower in SD902 under drought stress.

The ability of the antioxidant enzymes, including SOD, POD and GR in two varieties leaves were influenced by moderate drought (Fig. 2b, c, d, e)). Nevertheless, these antioxidant enzymes were upregulated in SD609 compared with SD902. The SOD, POD and GR activity in SD609 had increased by 26.4, 20 and 40.1%, respectively. However, the activities of SOD, POD and GR in SD902 increased by 8.1, 15.6 and 13.9%, respectively.

### Comprehensive proteome analyses of SD609 and SD902

**Fig. 4:**
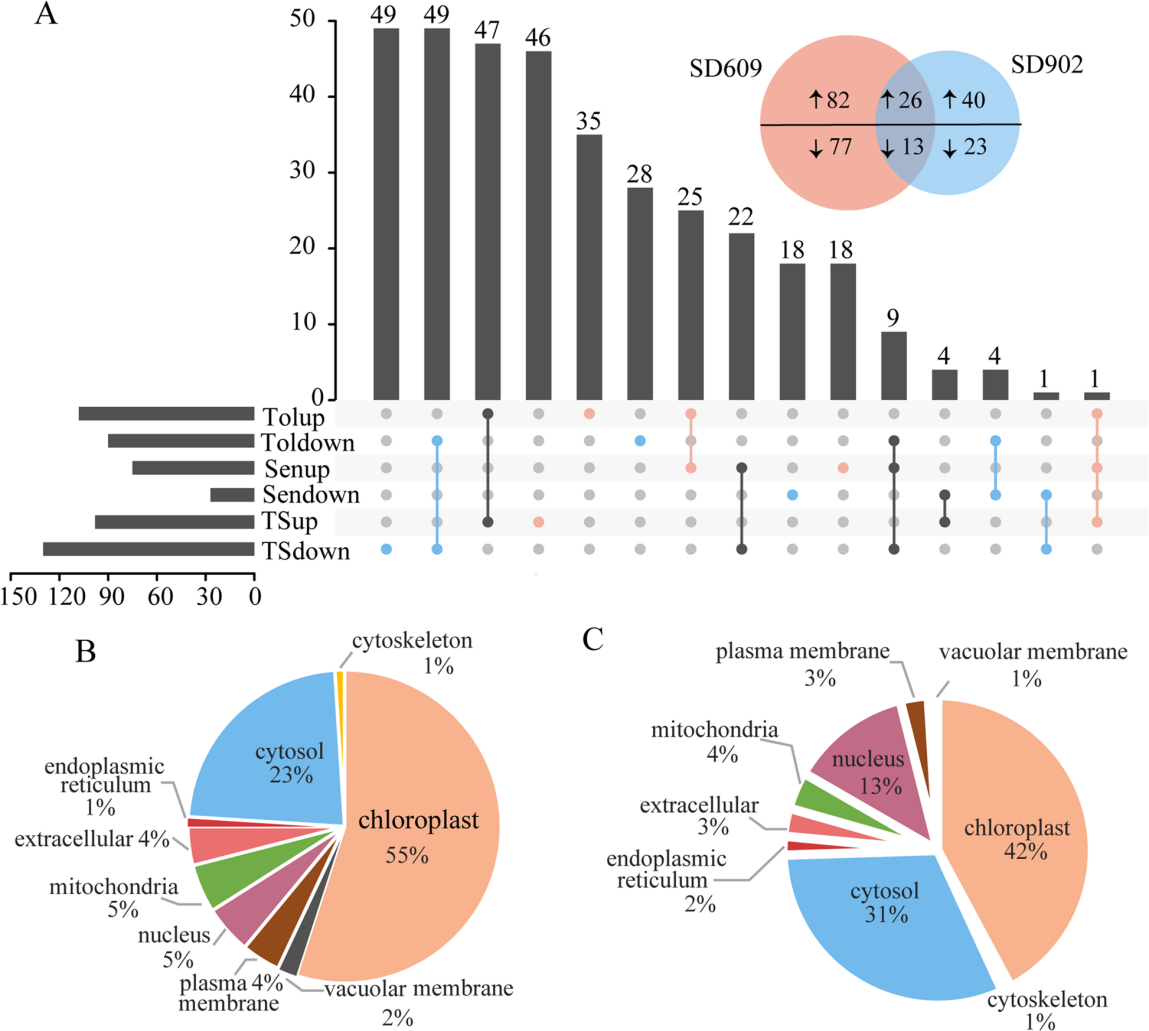
**A** UpSet plot between sets of proteins (Log_2_ FC > 1.5) in two maize varieties under drought stress. **B** Localizations of identified proteins in SD609. **C **Localizations of identified proteins in SD902

We used the iTRAQ approach and performed quantitative analysis on SD609 and SD902 to compare the differences in protein expression level between the control- and the drought-treated maize seedlings. We identified 5488 proteins with 1% false discovery rate (FDR) among 15,079 distinct peptides derived from 126,307 spectra. The search for proteins with fold change > 1.5 or < 0.67 (*P* < 0.05) returned 2559 differentially expressed proteins (DEPs) between the control and drought stressed SD609 and SD902. We then screened specific proteins based on the fold changes in their expression level. As shown in Fig. 4a, a total of 198 (108 upregulated and 90 downregulated) and 102 (66 upregulated and 36 downregulated) proteins significant changed (*P* < 0.05) in SD609 and SD902, respectively. Only 39 proteins overlapped between the two varieties, of which 26 were up-regulated and 13 down-regulated. Moreover, 159 proteins were unique to the drought-tolerant SD609, while 63 proteins were unique to the drought-sensitive SD902 (Additional Table 1).

**Fig. 5:**
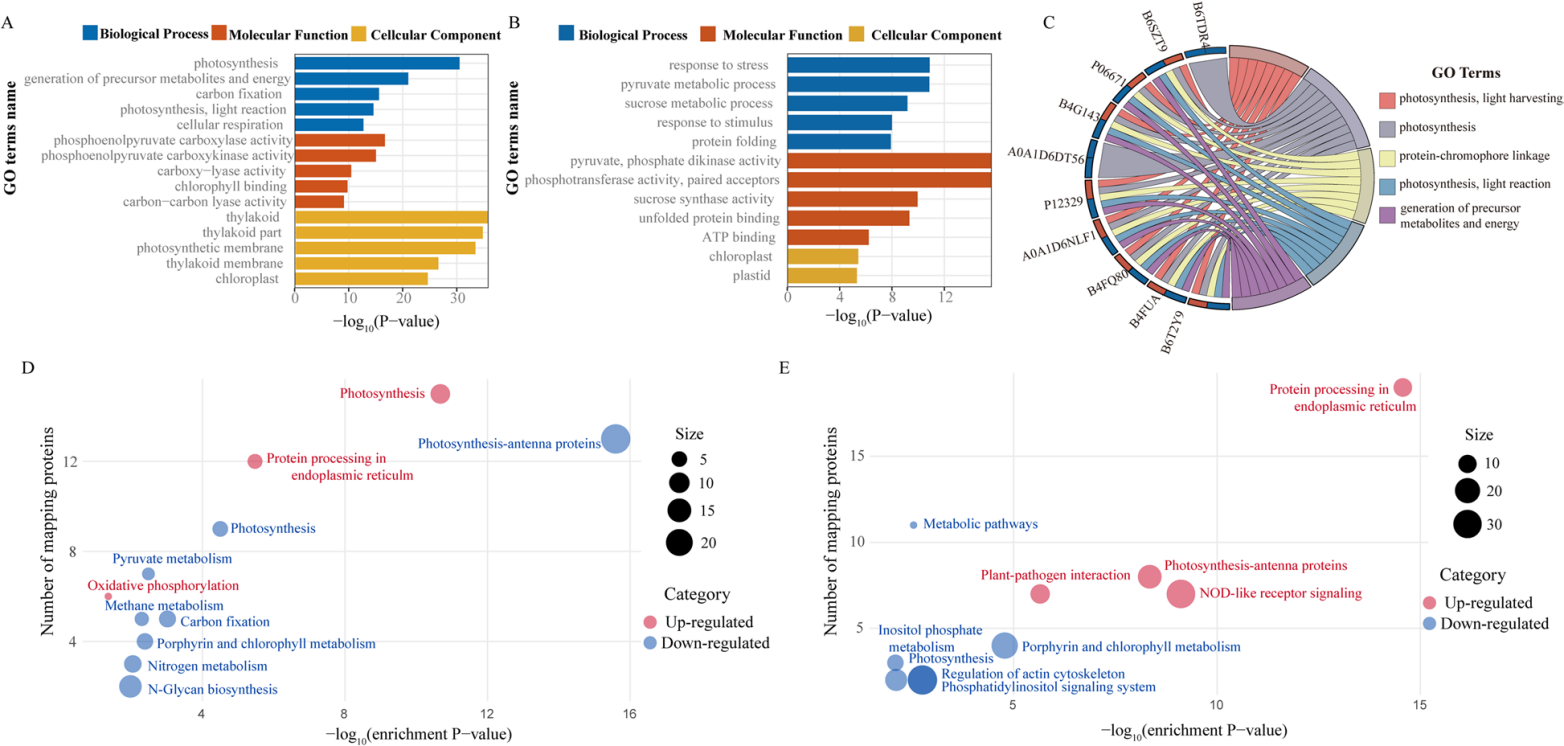
**A** Functional classification of unique differentially expressed proteins in SD609 under drought stress. **B** Functional classification of unique differentially expressed proteins in SD902 under drought stress. **C** Functional classification of shared differentially expressed proteins in two maize varieties under drought stress. **D** Bubble plot for KEGG pathway enrichment of DEPs in SD609. X-axis represents the number of mapping proteins and y-axis descripts the enrichment scores (−log_10_ [enrichment *P*-value]). The bubble size indicates the proteins expression in the KEGG pathways ((abs (log_2_fold change)) *2). **E** Bubble plot for KEGG pathway enrichment of DEPs in SD902

Next, we used the WoLFPSORT database to predict subcellular localization (Fig. 4b, c). Most of the DEPs were located in chloroplasts in SD609 (Fig. 4b) and SD902 (Fig. 4c). Hence, moderate drought stress mobilized numerous chloroplast proteins in both varieties. We performed gene ontology (GO) and Kyoto Encyclopedia of Genes and Genomes (KEGG) analyses to annotate the protein functions (Fig. 5). Among them, Fig. 5a and Fig. 5b respectively represented the GO terms of the specific DEPs in SD609 and SD902 induced by drought stress. The GO terms for the DEPs common to both varieties are shown in Fig. 5c. The GO data revealed that the specific DEPs in SD609 (Fig. 5a) were highly enriched in photosynthesis, whereas the specific DEPs in SD902 (Fig. 5b) were enriched in stress response. The biological processes of the DEPs shared by SD609 and SD902 involved photosynthesis and protein-chromophore linkage (Fig. 5c). The KEGG pathway analysis identified the metabolic pathways of the DEPs in SD609 and SD902 (Fig. 5d, e). The upregulated DEPs in SD609 (Fig. 5d) under drought stress were frequently associated with photosynthesis, while the downregulated DEPs were associated with photosynthesis-antenna protein and pyruvate metabolism etc. The protein processing endoplasmic reticulum and photosynthesis-antenna proteins KEGG pathways were relatively more frequent in the upregulated SD902 DEPs, whereas the porphyrin and chlorophyll metabolism were more frequent in the downregulated SD902 DEPs (Fig. 5e). Collectively, GO and KEGG enrichment results provided an overview showing that moderate drought caused the accumulation and suppression of distinct sets of proteins in SD609 and SD902.

### Photosynthesis-related DEPs observed in drought-tolerant SD609

#### Photosynthetic proteins in SD609

Photosynthesis is the main physiological process of plants and it rapidly responds to stress. A total of 36 DEPs in SD609 (10 up-regulated and 24 down-regulated) were related to electron transport (Additional Table 1). Five of these proteins (PSII repair protein PSB27-H1, Oxygen-evolving enhancer (OEE) protein 1-1, OEE1, OEE2-1 and PSII 11kD protein) were associated with PSII and increased under drought stress. The protein level of 14 light-harvesting Chl a/b binding protein complexes (LHCs) (such as LHCII, LHCP) were decreased by 0.22- to 0.55-fold. The protein Plastoquinol-plastocyanin reductase and plastocyanin in cytochrome b6/f complex were also up-accumulated by 1.7-fold relative to the control. Nine proteins (4 up-regulated and 5 down-regulated) annotated in PSI were altered under drought stress, of which the Ferredoxin 2 (FDX2), FDX5, two PSI reaction center subunit IV A proteins were increased, other proteins such as PSI subunit O, PSI-G, PSI-K as well as PSI-B were decreased. Moreover, four chlorophyll biosynthesis proteins were down-regulated induced by drought. In the dark (Calvin cycle) reactions of photosynthesis, nine phosphoenolpyruvate carboxylase (PEPC) proteins were decreased by 0.5- to 0.66-fold. The photosynthesis protein expression profiles suggest that the ETC structure and capacity were augmented in the drought-tolerant seedling leaves but diminished in the drought-sensitive seedling leaves under the same drought stress.

#### Reactive oxygen species (ROS) scavenging proteins in SD609

Plants have evolved an antioxidant defense system that comprises both enzymatic and non-enzymatic mechanisms and scavenges excess ROS under water deficit conditions. Here, we observed 16 ROS scavenging related proteins in SD609 (Additional Table 1). Relative SOD expression 9 was up-regulated by 2.4-fold, and five peroxidases were enhanced in SD609 under drought stress. Moderate drought stress also activated proteins associated with the ascorbate-glutathione (AsA-GSH) cycle and the thioredoxin-peroxiredoxin (Trx-Prx) pathway, such as glutathione reductase (GR), together with 2-Cys peroxiredoxin BAS1, thioredoxin M (TrxM).

#### Energy metabolism-related proteins in SD609

Consistent with the enriched GO biological process terms, mitochondrial electron chain and ATP synthesis-related proteins associated with energy metabolism were altered in SD609 under drought stress (Additional Table 1). Specifically, electron transport proteins such as the cytochrome c oxidase subunit and NADH-ubiquinone oxidoreductase B18 subunit were increased. Furthermore, two ATP synthase subunit proteins were also induced by drought. Their changes imply that SD609 enhanced energy production to cope with moderate drought stress.

#### Photosynthesis-related DEPs in SD902

The photosynthesis-antenna proteins pathway was significantly enriched in SD902 under water deficit conditions (Fig. 5e). However, more photosynthetic proteins were detected in SD609 than those SD902 (Additional Table 1). Eight DEPs (LHCs) involved in photoreaction were up-regulated by approximately 1.7-fold. Besides, 17 DEPs participating in photosynthesis were down-regulated. These 17 DEPs were grouped into three types: (i) five proteins related to Chl biosynthesis (for example, uroporphyrinogen decarboxylase and NADPH-protochlorophyllide oxidoreductase) were detected under drought stress. (ii) four Ferredoxin-NADP(H) oxidoreductase (FNR) protein, which catalyzes the electron transfer between NADP(H) and ferredoxin (Fd), were decreased in abundance. (iii) eight pyruvate, phosphate dikinase (PPDK) proteins involved in carbon fixation were significantly down-regulated in response to moderate drought stress. The down-regulation of most photosynthetic proteins may explain the changes in photosynthetic parameters of SD902 under drought stress. Besides, six sucrose synthase proteins involved in starch and sucrose metabolism were up-accumulated in SD902 under drought stress.

### DEPs common to both maize varieties

Thirty-nine proteins were shared by SD609 and SD902 (Additional Table 1) and included in the GO enrichment (Fig. 5c) of both datasets for photosynthesis and generation of precursor metabolites and energy. The most abundant photosynthesis-related proteins were LHCs and NADPH-protochlorophyllide oxidoreductase, in which the membrane proteins of LHCs bind chlorophyll and transfer energy to the reaction centers for photosynthesis. Of the 35 identified DEPs, most were upregulated in both varieties. As shown in Additional Table 1, the heat shock protein (HSP26) and other small heat proteins (sHSP), glutathione reductase, were up-regulated in both varieties under drought stress. By contrast, the LHCs was increased in SD902, but decreased in SD609.

### Protein-protein interaction network in SD609 under moderate drought stress

**Fig. 6:**
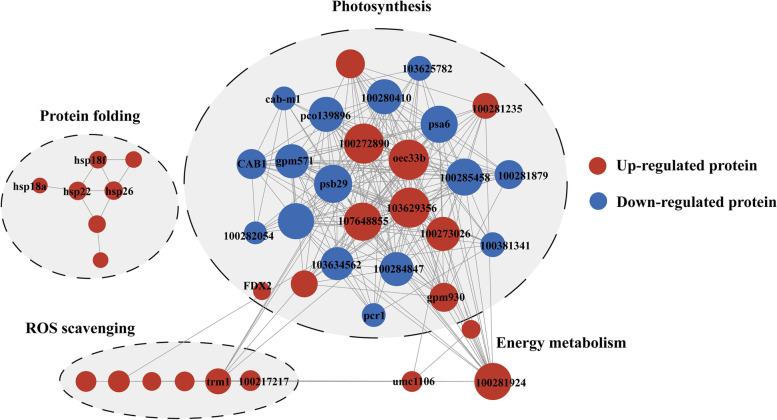
Analysis of the protein-protein interactions network in SD609 in response to drought stress

To predict the protein interactions and functional relations among DEPs, protein-protein interaction network analysis was performed with confidence scores > 0.5 to identify the interactions among specific DEPs in SD609 (Fig. 6). Four main interacting protein groups were identified in the network. Most of the proteins in these four clusters were upregulated and their functions were generally associated with photosynthesis, ROS scavenging, protein folding and energy metabolism. Most proteins in these clusters were increased, which displays the pivotal response of these proteins under drought conditions. Plastocyanin (103629356) interacted with 24 other proteins while OEE1 (100272890) was linked with 24 other proteins, such as PSI-K, cytochrome b6/f complex proteins and TRM1. Furthermore, ATP synthase subunit (100281924) interacted with 21 other proteins. These results indicate proteins involved in different metabolic pathways responded to drought stress by interacting.

### Expression levels of genes encoding DAPs in response to moderate drought

**Fig. 7:**
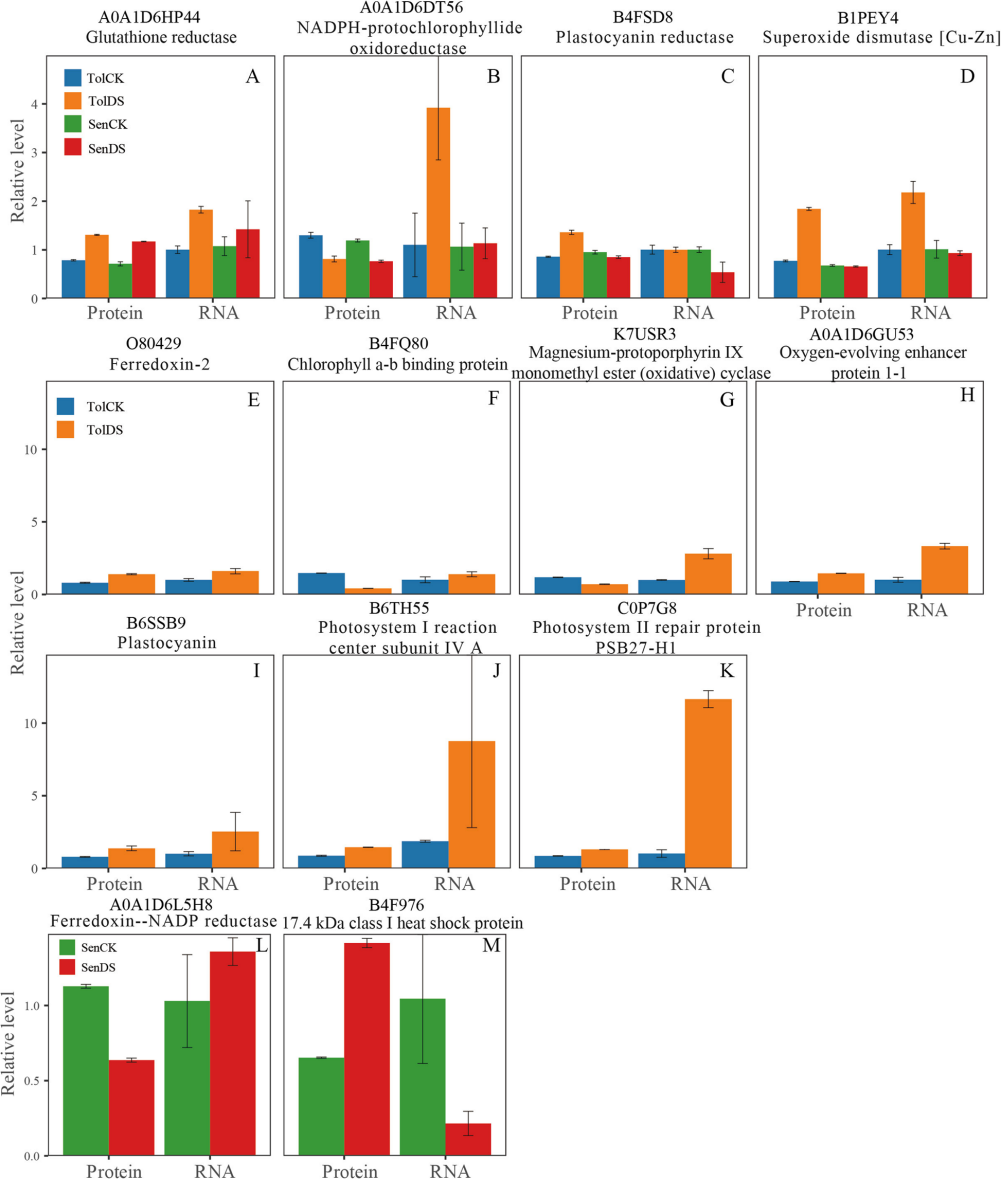
Confirmation of proteomic results by quantitative real-time PCR (qRT-PCR). **A**-**D** qRT-PCR analysis of four differentially expressed proteins shared between SD609 and SD902. **E**-**K** qRT-PCR analysis of seven differentially expressed proteins in SD609. **L**-**M** qRT-PCR analysis of seven differentially expressed proteins in SD902. Values were presented as mean ± SD (*n* = 3)

We used quantitative real-time PCR (qRT-PCR) to measure transcriptional expression of thirteen selected proteins. qRT-PCR results showed that the expression patterns of half the DEPs were coincided well with their corresponding coding genes. Of these 13 proteins, seven genes expression (Fig. 7a, d, e, h, i, j, k) were consistent with proteomic results. The expression patterns of the other six genes showed opposite trends with their homologous proteins. These results might be due to a time delay between mRNA and proteins or posttranscriptional and transcriptional regulatory mechanisms. Thus, most of the qRT-PCR results confirmed our proteomic results.

## Discussion

### Physiological responses of both maize varieties to moderate drought stress

The photosynthetic rate is usually affected by both stomatal limitations and non-stomatal limitations under drought stress [22]. Here, we found that the *P*_N_ and *g*_s_ of SD609 were higher than those of in SD902. As *C*_i_ had increased in both varieties, the observed decline in their net photosynthetic rates may have been caused by non-stomatal factors.

Plants have evolved complex mechanisms to contend with drought stress. We compared energy conversion between both maize varieties and found that foliar Y(II), Y(I), ETRII and ETRI were significantly lower in SD902 than SD609. Therefore, moderate drought reduced the LEF rate in both of them [12]. Y(NO) and Y(NA) in SD902 were significantly higher in SD902 than SD609. We speculate that the PSI and PSII photoinhibition had occurred in SD902. Y(CEF) was significantly higher in SD609 than SD902. These results indicated showed that the photosynthetic parameters of SD609 changes in the electron transfer between PSII and PSI more effectively than those of SD902. In SD609, the PSII and PSI activity levels were high, excitation pressure on PSII and PSI was mitigated, and the photosystems were protected.

Improving ROS scavenging is an adaptive response to drought stress [23]. SOD, POD and GR were significantly upregulated in SD609. Thus, SD609 effectively synthesized various antioxidant enzymes to counteract ROS production. These results generally aligned with those reported for a previous study on other plant species [10]. Thus, SD609 showed more well physiological performance under drought stress. All these protective effects help SD609 to survive under stressful conditions.

### Fine control of photosynthetic electron transport chain in tolerant variety

Photosynthesis involves the storage of light energy captured by photosynthetic electron transport reactions and the transfer of this energy to the Calvin-Benson cycle for carbon reduction [24]. Photosynthesis is highly sensitive to water deficit. The electrons released from water are transferred from PSII to PSI via the plastoquinone (PQ) pool, Cytb6f, and plastocyanin (PC) to reduce ferredoxin and NADP+. This electron transport pathway is known as linear electron flow (LEF). Moreover, the cycling electron flow (CEF) around PSI passes through the Cytb6f complex, generates ΔpH, and drives ATP synthesis without concomitant NADPH generation [25].

The GO and KEGG enrichment analyses showed that photosynthesis was the process most significantly affected by drought stress in SD609 and that drought induced Psb27, OEE1-1, and OEE2-1. Psb27 is a component of the PSII complex and participates in its assembly and repair [26], RNA-Seq disclosed that Psb27 was upregulated in pearl millet under drought stress [27]. OEE1 expression might be the rate-limiting step in PSII subunit assembly [28]. Our data suggested that maintaining PSII capacity by facilitating its assembly and recovery is a drought response mechanism. The cytochrome b6-f complex protein mediates electron transfer between PSI and PSII, controls state transitions in the thylakoid membrane, and governs the CEF around PSI [29]. Plastocyanin accepts electrons from cytb6f and transfers them to Fd via PSI. Thence, LEF may proceed from water to NADP^+^ via FNR to produce NADPH which enters the Calvin-Benson cycle. Alternatively, LEF might enter the CEF pathways and the electrons are transferred back to the PQ. This pathway may protect the photosynthetic mechanism against environmental stressors [30]. Corresponding with previous studies [31–33], our research demonstrated that drought upregulated Cytb_6_f complex protein, plastocyanin, and Fd proteins participating in CEF. These findings aligned with the Y(CEF) measurements for SD609 (Fig. 3e). Nevertheless, the LHC and the chlorophyll biosynthesis proteins capturing and transferring light energy to the PSII reaction center were downregulated in SD609 and upregulated in SD902. These responses might indicate that the PSII thylakoid membrane complexes are vulnerable to drought stress. This hypothesis would be consistent with the observed reductions in photochemical energy conversion efficiency by PSII and PSI (Y(II) and Y(I)) under stress conditions (Fig. 3a and b). Downregulation of these proteins suggests a regulatory mechanism for drought resistance in SD609. Limiting light absorption by reducing the LHCP and Chl levels could downregulate electron transport and decrease ROS production under stress conditions. This mechanism was corroborated by previous studies [34, 35], However, opposing results have also been reported [36] and the actual mechanisms remain to be clarified. The final step in the LEF transfers electrons from Fd and reduces NADP^+^ to support carbon fixation [37]. Whether or not FNR is directly involved in CEF, it partitions electrons between LEF and CEF [38, 39]. The observed reductions in FNR level in SD902 indicated that drought stress diminished photosynthetic electron transfer efficiency, certain fluorescence kinetics parameters, and the photosynthetic rate in SD902 compared with SD609 under drought stress. Thus, regulation of electron partitioning between the cyclic and linear pathways plays a key role in plant adaption to drought stress [38]. The results of the present study imply that appropriate PSII repair and CEF activation may minimize energy loss caused by reduced light absorption efficiency in SD609 in response to moderate drought stress.

Drought stress also influences the capacity of carbon assimilation in photosynthesis [40]. In C4 plants such as maize, PEPC catalyzes the conversion of phosphoenolpyruvate to oxaloacetic acid in the presence of HCO_3_^-^. PEPC forms part of a CO_2_ pump and provides oxaloacetic acid to the tricarboxylic acid cycle (TCA) [41]. A previous study concluded that under drought stress, malate inhibits PEPC [42]. PPDK catalyzes phosphoenolpyruvate regeneration in the C4 carbon fixation pathway [43]. Our findings revealed that PEPC and PPDK were substantially downregulated in SD609 and SD902, respectively, to enable them to contend with drought stress. These mechanisms were true for both maize and wheat [43, 44]. The observed reductions in PPDK level in SD902 might be associated with increased plant susceptibility to drought because PPDK participates in the production of NADPH which is an important component of various cell antioxidant and osmoprotectant mechanisms. Thus, downregulation of carbon fixation enzymes might explain the observed differences in photosynthetic rate between SD609 and SD902 under moderate drought stress.

### ROS scavenging pathway is activated in SD609 under moderate drought stress

Plants exposed to drought stress generate abundant ROS. They function as signaling molecules regulating numerous physiological processes, disturb intracellular redox balance, and cause oxidative cellular damage [45, 46]. Excess ROS scavenging or detoxification is achieved by an efficient antioxidant system comprising nonenzymatic (ascorbic acid (AsA) and glutathione (GSH)) and enzymatic (SOD, CAT, ascorbate peroxidase (APX), and monodehydroascorbate reductase (MDHAR)) antioxidants [23]. Of the 16 antioxidant-related proteins in SD609, CuZn-SOD was the first-line defense as it converts O2- to H_2_O_2_. CuZn-SOD overexpression enhances tolerance to various environmental stressors [47]. In plant cells, H2O2 elimination depends mainly on POD, CAT, peroxiredoxin (Prx), and other enzymes in the AsA-GSH cycle [48, 49]. The POD-related proteins were significantly upregulated in SD609 under drought stress. This discovery aligned with a previous report [50]. The thioredoxin reductase (Trx)-Prx pathway is a key antioxidant system in plants. Prx converts H_2_O_2_ to water and alcohols through Cys [51, 52]. TrxM is a member of the Trx family that removes ROS from chloroplasts and regulates Prx activity. Our results demonstrated that the Trx-Prx pathway alleviated the oxidative damage caused by drought stress in SD609. GR reduces GSSG to GSH and maintains the GSH pool in the AsA-GSH cycle. Glutathione S-transferase (GST) might play an important role in scavenging lipid hydroperoxide (LOOH) [46]. Thus, moderate drought stress could initiate the AsA-GSH and GST pathways and reduce oxidative damage in SD609.

### Pathways common to both maize varieties in response to drought stress

Dehydration stress also influences normal plant protein quantity and quality and induces stress-related proteins including HSPs [53]. We found that the various classes of heat shock proteins (HSPs) were induced in both SD609 and SD902 in response to drought stress. Therefore, the HSP expression patterns were genotype-specific [53]. HSP90, HSP70, and sHSP were induced to prevent cellular damage in SD902 whereas most sHSPs were upregulated in SD609 under drought stress. The members of each HSP class have specific functions. Nevertheless, cooperation among various HSP networks is a central principle of the integrated HSP machinery [54]. HSPs prevent protein aggregation and use ROS as signal molecules under stress conditions. HSPs are major stress-responsive proteins that may also participate in cross-talk with other mechanisms and function synergistically with other components to decrease ROS damage [55]. Future research should investigate the crosstalk between HSPs and other stress response mechanisms to elucidate stress tolerance in maize.

### Proposed molecular model of drought-tolerant maize varieties

**Fig. 8:**
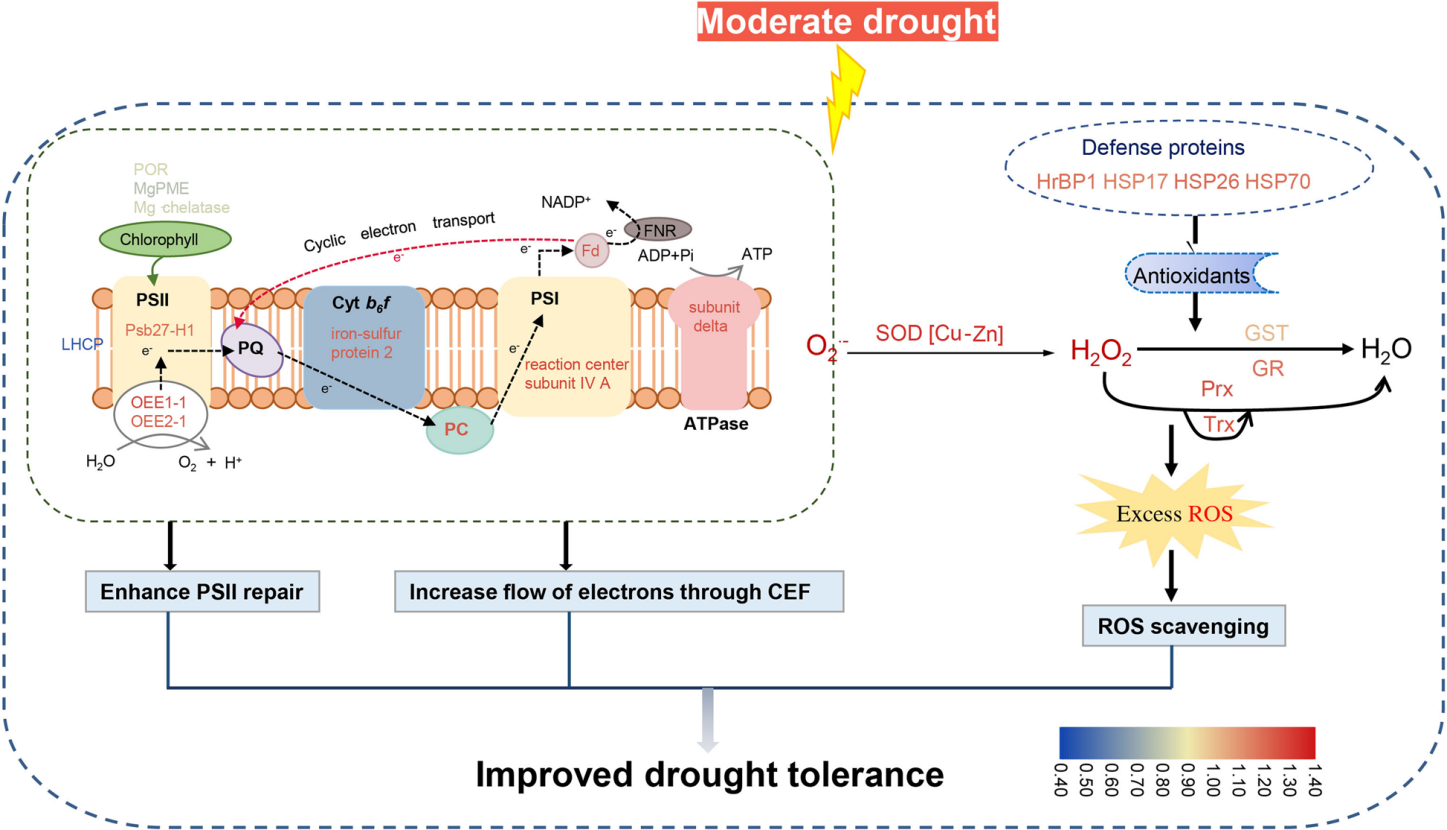
Model of proteins mechanisms in SD609 under moderate drought stress. The expression pattern of each protein is shown in box with colors

Based on the photosynthetic parameters and the drought-responsive proteins in SD609, our findings indicated that when maize leaves with different degrees of drought tolerance are subjected to water deficit, their protein fraction profiles change differently. In SD609, most of the proteins implicated in foliar photosynthesis and ROS scavenging were upregulated. This change was related to the concomitant occurrence of complex alterations in energy metabolism and the re-establishment of homeostasis. In contrast, only a few proteins were altered in SD902 in response to drought stress. A model of the responses of the photosynthetic electron transfer proteins to drought stress in SD609 is shown in Fig. 8. Drought stress downregulated chlorophyll biosynthesis and LHC proteins which, in turn, suppressed photochemical reactions and photosynthetic electron transport. However, PSII assembly, cytb6f, and PSI proteins accumulated under moderate drought stress to repair and stabilize PSII, increase the CEF around PSI, and enhance drought tolerance in SD609. Drought stress also activated the ROS scavenging system, augmented NADPH and ATP production, and facilitated protein folding. Similar results were reported for *Brachypodium distachyon* under H2O2 stress [56] and ginger under drought and shading conditions [57].

## Conclusion

Our physiological data suggested that the performance of the drought-tolerant maize variety (SD609) was superior to that of the drought-sensitive maize variety (SD902) under moderate drought. The former had relatively enhanced photoprotection and ROS scavenging mechanisms. The proteomics analysis validated the physiological data and demonstrated that the high drought tolerance of SD609 was associated with: (i) high photochemical efficiency and a strong photoprotective mechanism, (ii) an efficient antioxidant system and upregulated ROS-scavenging enzymes, and (iii) modulated protein metabolism preventing protein aggregation. In contrast, the sucrose and protein metabolism levels in SD902 did not suffice to enable the plant to contend with moderate drought. The qRT-PCR analysis validated the iTRAQ sequencing data. Overall, our results showed a clear relationship between the physiological mechanisms and the molecular events that occur in maize under drought stress and identified a list of candidate proteins implicated in drought tolerance that merit further investigation.

## Methods

### Plant materials and treatments

No permits were required to collect the plant materials. The drought-tolerant Shaandan609 (91227 x Chang7-2) and the drought-sensitive Shaandan902 (K22 × K12) commercial maize (*Zea mays.* L) hybrids were obtained from Shaanxi Dadi Seed Co. (Shaanxi, China). The maize seeds were grown in plastic pots (diameter 26 cm × height 38 cm) filled with 18 kg air-dried soil containing 1.62% organic matter and 0.064% total nitrogen [58]. All experiments were conducted in a greenhouse at the Northwest A&F University, Shaanxi, China in mid-May 2019. The average daytime and nighttime temperatures were 35 °C and 30 °C, respectively, the relative humidity was 50–60%, the day/night photoperiod was 16 h/8 h, and the maximum illumination intensity was ~ 2000 ± 50 mmol m^− 2^ s^− 1^. The soil moisture was maintained at 80% field capacity for 4 wks. Plants at the six-leaf stage were divided into two different watering treatments. The first was a well-watered control treatment (CK) wherein the plants were watered daily to maintain the soil water content (SWC) at 80%. The second was a drought-stress treatment (DS) in which the plants were irrigated to 50% SWC. The soil water status was monitored every evening to evaluate soil moisture content. After 7 d, the top third of the fully expanded leaves were selected to determine the photosynthetic parameters between 09:30 h and 11:30 h Beijing time. There were six replicates. The foliar relative water content (RWC) was measured for the third of the fully expanded leaves according to a standard method [58]. The leaves were subjected to fluorometric analysis, sampled, and stored at − 80 °C until the subsequent enzyme assays and proteome analyses. There were three biological replicates per treatment.

### Fluorescence parameter and enzyme activities determination

The energy conversion efficiency levels of PSII and PSI and the CEF activity were measured with a saturation-pulse Dual-PAM-100 (Heinz Walz, Effeltrich, Germany) using the top third of the fully expanded leaves according to Zhou et al. [12]. The leaves were dark-adapted for 30 min. The following parameters were assessed: maximum quantum yield of primary PSII photochemistry [F_v_ / F_m_ = (F_m_ - F_0_) / F_m_], the effective quantum yield of PSII photochemistry [Y(II) = (F_m_′ - F_s_') / F_m_′], The quantum yield of PSI [Y(I) = (P_m_′ - P) / P_m_], the quantum yield of non-regulated energy dissipation of PSII [Y(NO) = F_s_' / F_m_], the quantum yield of non-photochemical energy dissipation due to the acceptor side limitation [Y(NA) = (P_m_ - P_m_′) / P_m_], the quantum yield of non-photochemical energy dissipation due to the donor side limitation [Y(ND) = 1 - P700red], non-photochemical quenching [Y(NPQ) = (F_m_ / F_m_′) - 1], the ratio between the electron transport rate around PSII (ETRII) and PSI (ETRI): ETRI = Y(I) × PPFD× 0.85 × 0.5, ETRII = Y(II) × PPFD× 0.85 × 0.5, where 0.85 represents the leaf absorbance and 0.5 is the proportion of absorbed light energy allocated to PSI or PSII. The cyclic electron flow value (CEF) was estimated as ETRI-ETRII [32]. All measurements were conducted between 09:30 h and 11:30 h Beijing time.

To measure the antioxidant enzyme activity, each 0.3 g frozen leaf samples was ground in 10 mL of pre-cooled 100 mM phosphate buffer (pH 7.8), and the homogenate was centrifuged at 4000 g and 4 °C for 20 min. Superoxide dismutase (SOD, EC 1.15.1.1) activity was assayed using the method of Beauchamp and Fridovich [59]. Enzyme activity was detected by spectrophotometry at 560 nm. Peroxidase (POD, EC 1.11.1.7) were measured with guaiacol by monitoring the absorbance at 470 nm [60]. Glutathione reductase (GR: EC 1.6.4.2) activity was measured at 340 nm according to the method described by Grace and Logan [61] .

### Protein extraction, trypsin digestion and iTRAQ labeling

Protein extraction was performed according to a previously published method, with slight modification [62]. Briefly, the samples were ground in liquid nitrogen, transferred to 5-mL centrifuge tubes, sonicated thrice on ice with a high-intensity ultrasonic processor (Ningbo Scientz Biotechnology Co. Ltd., Ningbo, Zhejiang, China), and lysed with lysis buffer (8 M urea, 2 mM EDTA, 10 mM dithiothreitol (DTT), and 1% protease phenylmethylsulfonyl fluoride (PMSF; Beyotime Biotechnology, Shanghai, China). The lysate was centrifuged at 12,000×g and 4 °C for 30 min. The protein level in the supernatant was quantified with a bicinchonic acid (BCA) kit according to the manufacturer’s instructions.

For trypsin and iTRAQ labeling, the protein solution from each sample was reduced with 10 mM DTT at 37 °C for 30 min, alkylated with 25 mM iodoacetamide at room temperature in the dark for 15 min, digested with a 1:50 trypsin:protein mass ratio overnight, and digested again with a 1:100 trypsin:protein mass ratio for 4 h. The digested samples were incubated at room temperature for 2 h, pooled, desalted, dried by vacuum centrifugation, and labeled with iTRAQ reagent according to the manufacturer’s instructions.

### HPLC fractionation and LC-MS/MS analysis

The iTRAQ-labeled peptides were fractionated by high-pH, reverse-phase HPLC fitted with a Waters Bridge Peptide BEH C18 column (130 Å; 3.5 μm; 4.6 mm × 250 mm; Waters Corp., Milford, MA, USA). The peptides were distributed into 60 fractions using a 2–98% acetonitrile gradient over 88 min. They were then reconstituted into 12 fractions and concentrated by vacuum centrifugation. The tryptic peptides were dissolved in 0.1% (v/v) formic acid and loaded onto a reversed-phase analytical column. The eluent was a 0.1% (v/v) formic acid gradient increasing in the range of 5–45% over 58 min and rising to 80% in 2 min. The flow rate was 300 nL/min.

The peptides were subjected to the NSI source for LC-MS/MS analysis performed on a QExactive HF coupled to UPLC. The m/z range was 400–2000 for full scan and the resolution for the intact peptides was 60,000. The ion fragments were detected at 15,000 resolution. The 15 most intense precursors were selected for the subsequent decision tree-based ion trap higher energy collision-induced dissociation (HCD) fragmentation. The collision energy was 27% above the 1E5 threshold ion count in the MS survey scan and there was 20.0 s dynamic exclusion. Full width at half maximum (FHMW) at 400 m/z was used and coupled with an automatic gain control (AGC) setting of 1E6 ions and fixed first mass at 100 m/z.

### Protein identification and quantification

The MS/MS raw data were converted to .mgf file format with Mascot v. 2.3.02 (http://www.matrixscience.com/mascot_support_v2_3.html). Trypsin was selected enzyme and a maximum of two missing cleavages was permitted. Carbamidomethylation (C) was set as a fixed modification and oxidation (M) and acetylation in N-Term were set as variable modifications. The searches were performed using a 20-ppm peptide mass tolerance and a 0.05-Da product ion tolerance. The false discovery rate (FDR) was 0.05.

Proteins that differed between the drought-stressed and unstressed plants with fold change > 1.50 or < 0.67 (*P* < 0.05) were defined as significantly differentially expressed proteins (DEPs). Gene ontology (GO) and Kyoto Encyclopedia of Genes and Genomes (KEGG) pathway enrichments were performed using agriGO (http://systemsbiology.cau.edu.cn/agriGOv2/) and the KEGG database (https://www.genome.jp/kegg/), respectively. Only GO terms or KEGG pathways with *P* < 0.05 were defined as statistically significant. Protein interaction networks were constructed with the STRING database (https://string-db.org/) and Cytoscape v. 3.7.2 (https://cytoscape.org). Proteins with a combined score > 0.5 were interacting. Protein subcellular localization was predicted with WoLFPSORT (https://www.genscript.com/psort/wolf_psort.html).

### qRT-PCR

Thirteen candidate differentially expressed genes (DEGs) in the samples were selected to verify the iTRAQ results by qRT-PCR. The gene-specific primers used in this assay are listed in Table S2. The gene with ID No. GRMZM2G046804 was the internal control. Relative gene expression levels were calculated by the 2^-ΔΔCt^ method [63].

### Statistical analysis

The physiological assay and qRT-PCR results were analyzed with SigmaPlot v. 11.0 (Systat Software Inc., San Jose, CA, USA). Significant differences between the controls and treatments were determined by Tukey’s tests at a significance level of *P* < 0.05. Bioinformatic analysis was performed and graphics were plotted with R 3.6 (R Core Team, Vienna, Austria).

## Supplementary Information

**Additional file 1: Table S1.** Drought-responsive maize seedling leaf proteins observed specifically in tolerant variety SD609 and sensitive variety SD902, respectively; Differentially expressed proteins shared by two varieties under drought stress. **Table S2.** Corresponding genes of drought-stress responsive maize leaf identified proteins specific primers sequence.

## Data Availability

The mass spectrometry proteomics data have been deposited to the ProteomeXchange Consortium via the PRIDE partner repository with the dataset identifier PXD028508. After the data have been published, the access connection in ProteomeXchange will be: htp//proteomecentral.proteomexchange.org/cgi/GetDataset?ID=PXD028508.

## Abbreviations

POR: NADPH-protochlorophyllide oxidoreductase
MgPME: Magnesium-protoporphyrin IX monomethyl ester (oxidative) cyclase
Mg-chelatase: Mg-protoporphyrin IX chelatase
LHCP: Chlorophyll a-b binding protein
Psb27-H1: Photosystem II repair protein PSB27-H1 chloroplastic
OEE1-1: Oxygen-evolving enhancer protein 1-1 chloroplastic
OEE2-1: Oxygen-evolving enhancer protein 2-1 chloroplastic
PQ: Plastoquinol
Cyt *b*_*6*_*f*: Cytochrome b_6_/f complex
PC: Plastocyanin
Fd: Ferredoxin
FNR: Ferredoxin-NADP oxidoreductase
CEF: Cyclic electron flow
HrBP1: Harpin binding protein 1
HSP17: 17.0 kDa class II heat shock protein
HSP26: Heat shock protein 26
HSP70: Heat shock 70 kDa protein 14
SOD [Cu-Zn]: Superoxide dismutase [Cu-Zn] 4A
GST: Glutathione transferase
GR: Glutathione reductase
Prx: Thioredoxin-dependent peroxiredoxin
Trx: Thioredoxin family protein
RWC: Relative water content
